# Automatic planning of head and neck treatment plans

**DOI:** 10.1120/jacmp.v17i1.5901

**Published:** 2016-01-08

**Authors:** Irene Hazell, Karl Bzdusek, Prashant Kumar, Christian R Hansen, Anders Bertelsen, Jesper G. Eriksen, Jørgen Johansen, Carsten Brink

**Affiliations:** ^1^ Laboratory of Radiation Physics Odense University Hospital Odense Denmark; ^2^ Philips Healthcare Fitchburg WI USA; ^3^ Philips Electronics India Ltd. Bangalore India; ^4^ Department of Oncology Odense University Hospital Odense Denmark; ^5^ Institute of Clinical Research, University of Southern Denmark Odense Denmark

**Keywords:** automatic, treatment planning, head and neck

## Abstract

Treatment planning is time‐consuming and the outcome depends on the person performing the optimization. A system that automates treatment planning could potentially reduce the manual time required for optimization and could also provide a method to reduce the variation between persons performing radiation dose planning (dosimetrist) and potentially improve the overall plan quality. This study evaluates the performance of the Auto‐Planning module that has recently become clinically available in the Pinnacle^3^ radiation therapy treatment planning system. Twenty‐six clinically delivered head and neck treatment plans were reoptimized with the Auto‐Planning module. Comparison of the two types of treatment plans were performed using DVH metrics and a blinded clinical evaluation by two senior radiation oncologists using a scale from one to six. Both evaluations investigated dose coverage of target and dose to healthy tissues. Auto‐Planning was able to produce clinically acceptable treatment plans in all 26 cases. Target coverages in the two types of plans were similar, but automatically generated plans had less irradiation of healthy tissue. In 94% of the evaluations, the autoplans scored at least as high as the previously delivered clinical plans. For all patients, the Auto‐Planning tool produced clinically acceptable head and neck treatment plans without any manual intervention, except for the initial target and OAR delineations. The main benefit of the method is the likely improvement in the overall treatment quality since consistent, high‐quality plans are generated which even can be further optimized, if necessary. This makes it possible for the dosimetrist to focus more time on difficult dose planning goals and to spend less time on the more tedious parts of the planning process.

PACS number: 87.55.de

## I. INTRODUCTION

A number of uncertainties and variations are present in radiotherapy such as absolute dose precision,^(1)^ delivery precision,^2^, ^3^, ^4^, ^5^ precision of calculated dose distributions,^6^, ^7^, ^8^, ^9^ and radioresponsiveness of the specific tumor and normal tissues.^10^, ^11^, ^12^, ^13^, ^14^, ^15^ Two of the largest variations within radiotherapy are the heterogeneity in target definition^16^, ^17^, ^18^ and the variation among treatment plans for a given geometry both intra‐ and interinstitutional.^19^, ^20^ Most treatment plans are likely to have sufficient dose coverage of the delineated targets, but large variations in dose to healthy tissues occur.

The dose distribution depends on the dose objective defined by the dosimetrist, typically in accordance with institution‐specific guidelines. However, even guidelines do not ensure an optimal dose distribution for the specific anatomy, since the lower achievable dose limit to an OAR for a specific patient is unknown. This is the reason why treatment plans are optimized for the individual patient by trained dosimetrists. Moreover, the treatment optimization is labor‐intensive work with a very large solution space, which makes it difficult to ensure that the clinical treatment plan is the optimal plan. Therefore, there is a need to automate the treatment planning optimization procedure both to reduce the amount of time spent on the optimization and, more importantly, to reduce the interdosimetrist variation.

If an automatically generated treatment plan of high clinical quality is available prior to manual optimization, it could serve as a quality reference and starting point for the specific treatment and thereby ensure a certain minimum quality. Furthermore, the automatic plans could potentially be a time‐saving tool during the treatment optimization, which would reduce one of the most tedious steps in the process. Sharing the optimization parameters between institutions could also provide a method to share knowledge and standardize plan quality.

Previous documented solutions with somewhat different approaches have shown the potential of automation of the planning process.^21^, ^22^, ^23^, ^24^, ^25^ The current study validates the performance of a prototype version of the Auto‐Planning module which recently has been productized for clinical use in the Pinnacle^3^ treatment planning system from Philips Healthcare (Fitchburg, WI).

## II. MATERIALS AND METHODS

The Auto‐Planning software was evaluated by replanning 26 previously delivered clinical head and neck IMRT treatment plans of the oropharynx. The plans were delivered over the 12 months prior to the study. The plans were created in accordance with the Danish Head And Neck Cancer Groups guidelines (DAHANCA ‐ Version 2004), and each dose plan included three dose levels of 50 Gy, 60 Gy, and 66 or 68 Gy in 33 treatment fractions with a simultaneous integrated boost technique.

In the Auto‐Planning software, a template of configurable parameters known as a Technique (details in Appendix A) can be defined for each treatment protocol. The Techniques include definition of beam parameters and planning goals. The Auto‐Planning module uses the Technique definition to iteratively adjust IMRT planning parameters to best meet the planning goals. The Technique was defined according to local standards, including prioritization between target coverage and dose to organs at risk. The Technique definition was based on five additional pilot patients independent of the 26 study patients.

Each of the 26 treatment plans were replanned with Auto‐Planning without knowledge of the clinically delivered treatment plans and without any dosimetrist postoptimization of the treatment plans. The only input to the replanning was the delineations of planning target and organs at risk and the positioning of the isocenter.

Quantitative dosimetric evaluation of the performance of the treatment plans was performed on dose volume histograms (DVH) extracted from the planning system. CT scans had a slice thickness of 3 mm and in plane voxel size of 1 mm×1 mm. The dose plans were calculated using the Pinnacle^3^ collapsed cone algorithm with a dose grid resolution of 3 mm. Specific DVH values, as well as the overall shape of the DVH, was compared using average DVHs of the two types of treatment plans.

The average DVH was calculated for each type of treatment plan as the average of the patient‐specific DVH values at each dose level. To specify dose regions for which statistically significant differences exist, a probability curve as in Bertelsen et al.^(26)^ was calculated. In short, the probability curve is a Wilcoxon matched‐pair, signed‐rank test performed at each dose level. Individual values of the curve are not strict statistical tests since the test is performed multiple times and on values that are not mutually independent. The probability curve is, therefore, primarily a tool that indicates regions for which the average DVHs deviate significantly.

The DVH analysis was performed for all target volumes, as well as for parotid gland, submandibular gland, and spinal cord. To evaluate dose to other normal structures than the delineated, a DVH evaluation of all healthy tissues outside the PTV was performed. In contrast to the previous DVH evaluations, this evaluation was performed in absolute volume to compensate for a difference of the CT scanned volume of each patient (relative values would depend on the scanned volume).

The dosimetric evaluation was extended with a blinded clinical evaluation of the treatment plans. Two senior head and neck radiation oncologist independently scored the treatment plans on a categorical scale from 1 to 6 (1=bad and 6=good). The scoring was performed for target coverage, sparing of healthy tissues, and an overall assessment of the treatment plan. Finally, based on the clinical evaluation, the radiation oncologists selected the plan they would favor for clinical treatment. The radiation oncologist had access to all clinical information and diagnostic scans. The evaluation was performed similar to the clinical procedure used for evaluation of two different proposals for a given clinical treatment. All information related to the production of the plans was blinded and the treatment plans were presented for evaluation in random order.

All statistical tests were made by Wilcoxon matched‐pair, signed‐rank test using a significance level of 5%.

## III. RESULTS

**Figure 1:**
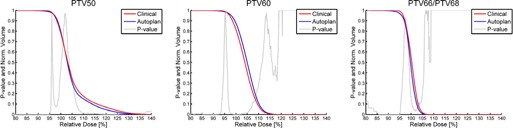
Mean DVHs of the target volumes for the clinical (red) and auto (blue) plans normalized to the prescribed doses for each PTV. P‐value curves are shown in gray.

Average DVH comparison between automatic and the clinical delivered plans for the targets is shown in Fig. 1. In accordance with ICRU 83,^(27)^ PTV50 includes PTV60 and PTV 66/68 causing the long tail towards high doses. Likewise, PTV60 contains PTV66/68. For PTV50, the autoplans had a small, but statistically significant, lower dose than the clinical plans below 95% of the 50 Gy and also above ∼110% of the 50 Gy.

The PTV60 had a similar pattern as the PTV50 below ∼92% of 60 Gy. A slightly higher average dose for the autoplans was also observed, while no differences were seen for higher doses. For the highest dose level, PTV66/68, the trend of a small, but statistical lower, dose below 95% of prescribed dose was observed for the autoplans, while no other differences were found.

The observations from Fig. 1 are reflected in Table 1 which shows selected DVH parameters. The difference in steepness of the DVH for PTV50 is statistically significant, with the automatic plans having the steeper slope. Also, the minimum dose was different for the two types of plans, with the clinical plans having a slightly higher minimum dose. Nevertheless, for both types of plans, the average DVH covers the PTV50 with the 95% isodose lines for at least 98.5% of the target which meets the ICRU 83 recommendation,^(27)^ as well as the 2013 DAHANCA guidelines and QA evaluations.^(28)^ For PTV60, the only statistically significant differences observed were a slightly higher dose level for the automatic plans (Table 1). For the highest dose level, the automatic plans produce more conformal dose distributions (Conformity Index CI 95%), but slightly less maximum and minimum dose.

**Table 1:** Mean values of standard DVH parameters for PTVs. Mean dose and $D_{V}$ are percent of the dose prescribed to the specific PTV. The standard deviations (SD) of the dose to PTVs are in percent points of the prescribed dose and $V_{D}$ are in volume fractions. CI 95% is defined as the ratio of the 95%‐isodose‐volume and the volume of the PTV. Uncertainties are reported as 1 SD, except for the median volume where ranges are reported. Statistically significant differences are shown in bold.

	*PTV50*			*PTV60*			*PTV66/68*		
*Parameter*	*Clinical*	*Auto*	*P*	*Clinical*	*Auto*	*P*	*Clinical*	*Auto*	*P*
Median volume (cm^3^)	266 (91‐323)			90 (36‐175)			80 (20‐264)		
Mean dose (%)	105.17±2.28	104.50±2.88	0.091	103.99±1.27	104.90±1.68	**0.034**	100.29±0.74	99.70±1.24	0.058
SD of dose to PTV (%)	6.98±2.15	5.92±1.52	<0.001	3.99±0.66	3.89±0.69	0.209	2.12±0.42	1.93±0.63	0.124
D_5%_‐D_95%_ (%)	23.15±7.19	19.48±5.37	<0.001	13.06±2.15	12.52±2.21	0.078	6.89±1.33	6.18±1.99	0.078
D_2%_ (%)	124.81±7.17	122.68±6.11	**0.006**	111.79±2.31	112.02±2.30	0.585	104.29±1.24	103.16±1.63	**0.012**
D_50%_ (%)	103.37±1.60	103.17±2.70	0.469	104.13±1.35	105.06±1.70	**0.023**	100.33±0.77	99.84±1.20	0.159
D_98%_ (%)	96.10±0.72	96.46±2.74	0.694	96.03±0.92	96.56±2.25	0.182	95.93±0.89	95.09±2.36	0.182
V_95%_	0.992±0.006	0.985±0.014	**0.001**	0.990±0.008	0.987±0.014	0.869	0.992±0.009	0.969±0.047	**0.002**
V_107%_	0.249±0.132	0.209±0.183	0.096	0.241±0.124	0.316±0.168	0.078	0.001±0.003	0.001±0.004	0.966
CI_95%_	1.48±0.15	1.42±0.23	1.000	1.64±0.29	1.65±0.15	0.844	1.74±0.20	1.28±0.17	**0.031**

**Figure 2:**
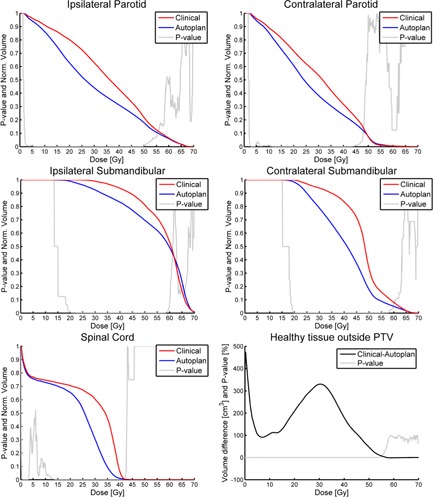
Mean DVHs of the OAR for the clinical (red) and auto (blue) plans. P‐value curves are shown in gray. Figure 2(f) show the average absolute volume difference in cm^3^ between the DVHs for the healthy tissue for the clinical minus the autoplans.

Average DVH curves for the organs at risk (OAR) are shown in Fig. 2. For all the delineated organs, the average DVH doses from the autoplans are either less than or equal to those of the clinical plans. There are no dose ranges for which the autoplans generated statistically significant higher doses to the delineated OAR.

To evaluate dose to nonde‐lineated healthy tissues, Fig. 2(f) shows the DVH difference for all tissues outside the PTV. The figure demonstrates the difference of the absolute volume irradiated above a specific dose level for the clinical plans minus the automatic plans. For all dose levels up to ∼55 Gy, the dose levels outside the PTVs were lower for the automatic plans. Above ∼55 Gy, the two types of plans were equal to each other.

To quantify the DVH differences and their variation, selected DVH metrics for the organs at risk are shown in Table 2.

**Figure 3:**
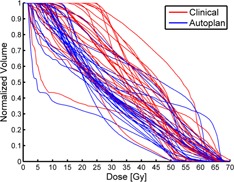
Pairs of DVH for ipsilateral parotid, illustrating the less variation of autoplans.

The overall result in Table 2 is as stated above, that the OAR receives less dose using the automatic generated plans than the previously delivered plans. Part of the observed dose differences was related to interdosimetrist variation. Figure 3 shows an example of all pairs of DVH for ipsilateral parotid, which illustrates that the autoplans are confined to a narrower region than the manual plans.

The evaluations by the oncologists are shown in Table 3.

Except for Patient 16, the main finding is that the qualities of both types of plans are good. Patient 16 failed to meet the maximum dose goal to the spinal cord which has a very high clinical priority, but for the other targets and organ delineations, the plan was evaluated as good. In terms of target coverage, the scores were not statistically significantly different between the two types of plans for any of the two observers; however, a p‐value of 0.079 for one observer could indicate a preference for the clinical plans in term of target coverage.

The clinical evaluation of the dose to the OARs was clearly in favor of the autoplans for both observers, which is consistent with the dosimetric observations in Fig. 2 and Table 2. For the overall evaluation of the plans, one observer is clearly in favor of the autoplans, while the other observer showed no statistically significant differences. In 94% of the cases, the overall score for the autoplans were as good as, or better than, the clinical plans. The last column of Table 3 shows the preferred treatment plans by the clinicians. The column reflects the overall plan score, and shows that the observer with a difference in the overall evaluation only selected automatic plans while the observer who did not find a significant overall difference selected the automatic plans and the clinical plans evenly.

**Table 2:** Mean values for doses to organs at risk. Uncertainties of all values are reported as 1 SD. Statistically significant differences are shown in bold.

	*Clinical*	*Auto*	^*p*^
*Spinal Cord*			
Mean (Gy)	26.03±5.13	21.13±4.55	<0.001
Max (Gy)	42.35±1.56	39.60±3.62	<0.001
D_20%_ (Gy)	40.50±1.45	36.23±3.60	<0.001
V_35Gy_	0.46±0.19	0.09±0.10	<0.001
*Submandibular Contralateral*			
Mean (Gy)	47.33±7.66	40.41±6.87	<0.001
V_30Gy_	0.94±0.20	0.79±0.19	<0.001
V_39Gy_	0.85±0.25	0.56±0.21	<0.001
V_60Gy_	0.08±0.16	0.05±0.12	0.074
*Submandibular Ipsilateral*			
Mean (Gy)	57.62±6.78	54.54±9.18	**0.001**
V_30Gy_	0.99±0.03	0.92±0.11	<0.001
V_39Gy_	0.94±0.12	0.84±0.20	<0.001
V_60Gy_	0.53±0.33	0.49±0.30	0.072
*Parotid Contralateral*			
Mean (Gy)	30.48±5.71	26.55±4.02	<0.001
V_5Gy_	0.96±0.07	0.94±0.08	**0.033**
V_26Gy_	0.60±0.19	0.46±0.11	<0.001
V_35Gy_	0.42±0.16	0.30±0.12	<0.001
*Parotid Ipsilateral*			
Mean (Gy)	35.54±6.84	29.73±5.57	<0.001
V_5Gy_	0.96±0.09	0.94±0.12	**0.007**
V_26Gy_	0.71±0.19	0.51±0.11	<0.001
V_35Gy_	0.52±0.16	0.37±0.12	<0.001

**Table 3:** Clinical evaluation of the auto and clinical plans on a score from one to six (1=bad – 6=good). The observers scores are shown in each columns separated by a slash. At the lower part of the table, mean values for both the individual observers and a combined score is shown together with a test of statistically significant differences between the score for auto and clinical plans. For Patient 16 please see note in text. Statistically significant differences are shown in bold.

	*Target Coverage*		*Organs at Risk*		*Overall Quality*		*Selected Plan*
*Patient*	*Clinical*	*Auto*	*Clinical*	*Auto*	*Clinical*	*Auto*	Clin=0; Auto=1
1	5/6	6/5	4/5	5/6	5/5	6/5	1/0
2	4/5	5/5	6/4	5/6	5/4	5/6	1/1
3	6/5	6/6	5/4	6/6	5/5	6/6	1/1
4	6/6	6/6	5/5	6/6	5/6	6/6	1/1
5	6/6	6/5	5/5	6/6	5/6	6/6	1/0
6	6/5	6/6	4/5	5/6	5/5	6/6	1/1
7	6/5	6/6	5/5	6/6	5/5	6/6	NA/1
8	6/6	6/5	5/5	6/6	6/5	6/5	1/0
9	5/6	6/5	5/5	5/6	5/6	6/6	1/0
10	6/5	5/6	4/3	6/5	4/3	5/5	1/1
11	6/6	6/6	4/5	6/6	5/6	6/6	1/1
12	6/6	6/6	4/5	6/6	5/6	6/6	1/1
13	6/6	6/5	4/5	6/6	5/6	6/6	1/0
14	5/6	5/5	4/5	6/6	5/6	6/6	1/0
15	6/6	6/6	4/5	6/6	5/6	6/6	1/1
16	6/6	5/5	4/6	6/1	5/6	6/1	1/0
17	5/5	5/6	4/4	5/6	NA/4	NA/6	1/1
18	6/6	5/4	4/5	5/5	4/6	5/5	1/0
19	5/6	6/6	4/6	6/6	5/6	6/6	1/0
20	5/6	5/6	4/6	6/6	5/6	6/6	1/1
21	5/6	6/5	4/6	6/6	5/6	5/6	1/0
22	6/6	6/6	5/5	6/6	5/6	6/6	1/1
23	6/6	6/5	5/5	6/6	5/5	6/5	1/0
24	6/4	5/3	5/5	6/6	6/4	6/4	1/0
25	6/6	6/5	5/5	6/6	5/6	6/6	1/0
26	6/NA	6/NA	4/NA	6/NA	5/NA	6/NA	1/NA
Mean Observ	5.65/5.68	5.69/5.36	4.46/4.96	5.77/5.72	5.00/5.40	5.84/5.52	1.00/0.48
Mean Total	5.67	5.53	4.71	5.75	5.20	5.68	0.74
P Observ	0.739/0.074		<0.001/0.001		<0.001/0.285		
P total	0.194		<0.001		<0.001		

## IV. DISCUSSION

For all 26 patients it was possible to produce automatic plans of high plan quality. Small differences in the PTV dose coverage but a significant reduction in dose to OAR between automatic plans and clinical plans indicate that the Technique parameters used in the current study are biased towards normal tissue sparing relative to the clinical practice. It is likely that another set of Technique parameters could be determined which would focus more on dose coverage. In addition, in the clinical release of the Auto‐Planning software, modifications have been made to enhance the priority of maximum dose constraints to OAR such as the cord, a need highlighted by the physicians during plan evaluation. No postoptimization of the plans was performed in the study to make a pure validation of the Auto‐Planning software. If needed or requested, in a clinical situation automatic optimized plans could be further optimized just as any manually created plan since the automatic plans are delivered in exactly the same format as manually created plans. Thus, the automatic plans can either be a high quality starting point for further manual optimization or an attempt to produce plans of clinical quality without further user intervention.

It is likely that the reduced dose to normal tissue of the sizes seen in Fig. 2 could be of clinical impact for the patients, while the small difference in tumor coverage will almost certainly have no clinical impact. However, without no definitive answer to which plan is the better in terms of tumor control and sparing of normal tissue (e.g., based on large randomized trials), the evaluation of the clinical quality of the plans will be somewhat subjective and depend upon individual views of the oncologist judging the plans. In the current study there are indications that the two oncologists have slight differences in their priorities. As stated in the results, both oncologists scores the OAR irradiation significantly better for the automatic plans compared to the manual plans, while none of the oncologists found statistically significant differences between the automatic and manual plans in terms of target coverage; although a p‐value of .074 for one of the oncologist indicates a favor of target coverage from the manual plans. These differences are also reflected in selection of treatment plan in which one oncologist only select the automatic plans while the other select an even mixture of automatic and manual plans. However, independent of the interoncologist variations, it is interesting to observe that in 94% of the cases (all except for one) the overall score given to the automatic plan is at least as high as the score for the manual plan, indicating that most of the plans could be used clinically without any user intervention. Only for one plan, one of the oncologists scored the automatic plan lower than the manual plan due to a violation of the maximum dose constraint of the spinal cord. With slight manual effort, this single treatment plan was later optimized to have an acceptable maximum spinal cord dose.

A prerequisite to achieve high‐quality automatic plans compared to manually created plans is obviously a sound optimization and tuning algorithm (the Auto‐Planning engine). However, the quality of the manual plans is obviously also of importance in comparison with the automatic plans (poor quality manual plan would favor automatic plans). At the time the manually created plans were made, all clinical head and neck treatment plans in the department were created using IMRT; thus the department was experienced in creating “high quality” plans manually. As a result, given the significant experience with IMRT in the department, the interdosimetrist variation should be limited. Nevertheless, as seen in Fig. 3, there is quite a variation in manually optimized plans, a variation which is less for the automatic optimization. It therefore seems likely that one of the advantages of automatic plans is a reduction of the interdosimetrist variation which is present even within departments that use IMRT extensively. The reason of the interdosimetrist variation is related both to limited time to create the plan and to lack of knowledge of how much the plan in reality can be optimized. For a manually created plan, it is difficult to know the extent an OAR can be spared prior to actual plan optimization. Therefore, objectives for organs at risk are typically set relatively loose initially, in order to ensure dose coverage of the target. Having obtained dose coverage of target, the next step is to reduce the dose to organs at risk as much as possible. In a busy clinic, it can be hard to ensure that all constraints on organs at risk have been tightened as much as possible. This issue could be reduced significantly if an initial “high quality” plan — e.g., an automatic generated plan — were available such that the dosimetrist could focus on fine tuning of the treatment plan.

Another potential benefit of Auto‐Planning could be a simple method to exchange planning knowledge and procedures between institutions since the Technique configuration of the Auto‐Planning software can easily be shared between institutions. This could help institutions with, for example, limited resources to quickly create IMRT or VMAT plans with similar quality as in more advanced institutions.

Most previous work on automating the planning process has built on knowledge of previously treated patients. One approach of extracting information from previously treated patients is utilizing the overlap volume histogram method, which measures the position of an OAR relative to the target.^19^, ^29^, ^30^, ^31^ Knowledge of overlap volume histograms from previously treated patients can be used to predict the likely achievable irradiation level of specific OARs. A few published solutions on automating the planning process have been documented,^21^, ^22^, ^23^, ^24^, ^25^ and all build on the Pinnacle^3^ planning system. The published systems did show the feasibility of automating the planning process. However, in terms of flexibility, it could be a potential issue that “knowledge based” approaches require a database of “high” quality plans for each protocol. Changes to planning techniques, prescriptions, OAR sparing goals, and contouring style could, if not implemented in a very smart way, require a new “high” quality database. Such a change could be quite labor‐intensive to implement clinically, and might not be as flexible to interchange between institutions. This issue might be addressed within “knowledge based” algorithms, but is not present in the Auto‐Planning solution evaluated in this study since it only relies on a small set of Technique parameters.

Finally, it should be mentioned that plan comparison studies are inherently difficult to perform since development of the treatment planning skill is continually ongoing within any department in order to optimize the treatments plans. Thus, if the current study was repeated, the results might be different since our dose planning team has learned new ways to improve the quality based on the results of this study. Similar statements could be made about the configuration of the automatic system. However, this does not change the fact that the current comparison between treatment plans that have been delivered clinically and the automatic treatment plans did show the autoplans to be superior at that time. Thus, the impact of Auto‐Planning seems likely to be a tool to increase the overall quality of dose planning, rather than a tool that could remove the need of manual optimization.

## V. CONCLUSIONS

Comparison of autoplans and previous delivered clinical plans showed only small dosimetric differences in target coverage, but significant reduction in dose to OAR for the autoplans. The blinded clinical evaluation of the plans showed that, for 94% of the evaluations, the autoplans were similar to or better than the clinical plans. Auto‐Planning software will, therefore, be able to reduce the manual time spend per treatment plan since the most of the plans could potentially be used clinically without further optimization. Perhaps more importantly, Auto‐Planning could be used as a high quality starting point for further plan optimization. This could increase the overall quality of the treatments and reduce the interobserver variation present in manually created treatment plans.

## ACKNOWLEDGMENTS

This work is related to AgeCare (Academy of Geriatric Cancer Research), an international research collaboration based at Odense University Hospital, Denmark. Research agreement with Philips Healthcare is acknowledged.

## Supporting information

Supplementary Material

Supplementary Material

Supplementary Material

## Appendix A. Description of Auto‐Planning.

This Auto‐Planning module simplifies the planning process through the use of templates called Techniques, and automatic optimization tuning methods called the Auto‐Planning engine (APE).

The user can define the following parameters in a Technique:

– Derived regions of interest (ROIs) (e.g., PTV or expanded cord)
– Placement of points of interest (POIs)
– Prescriptions
– Beam geometries, settings, and optimization options
– Prioritized optimization goals

A single selection creates a new plan based on the Technique settings and runs the APE. The APE tuning method maps the prioritized optimization goals defined in the Technique to optimization objectives. Multiple optimization loops are performed that iteratively adjust the optimization parameters to meet the goals and further drive down organ at risk (OAR) sparing with minimal compromise to the target coverage. This is achieved by using objectives specific to driving down OAR dose to the point it significantly affects target coverage and separate objectives to achieve the desired goals. Target conformality is automatically controlled by a system‐generated ring structure, and objectives and body dose is controlled by a system‐generated normal tissue structure and objectives. The objective dose and weight parameters are tuned using a proprietary method. Target uniformity is controlled by reducing hot and cold spots using system‐generated control structures and objectives similar to the process defined in the study by Xhaferllari et al.^(32)^

The input to a Technique is clinical goals (e.g., maximum dose, mean dose, and DVH constrains), as well as the pertinent parameters listed above. Optimization of a Technique is an iterative process in which Auto‐Planning is performed on test patients. If there are short‐comings in the autoplan results, the optimization goals in the Technique are adjusted to account for them and saved. In the current work, the optimization of the Technique was performed on separate patients than those included in the study, and finalized before autoplanning was performed on any of the patients included in the current study.
