# Supplementary figures and images for: Automatic planning of head and neck treatment plans
Source: J Appl Clin Med Phys. 2016 Jan 8;17(1):272–82. doi: 10.1120/jacmp.v17i1.5901 (PMC5690191; doi:10.1120/jacmp.v17i1.5901)

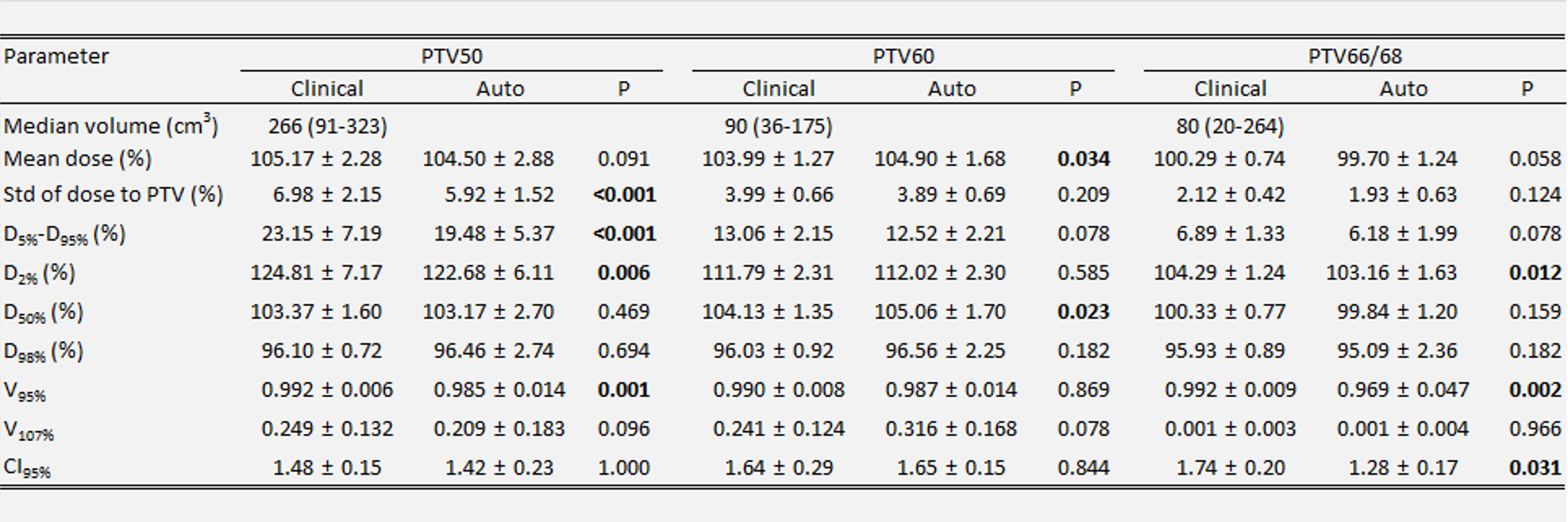

Supplement: Supplementary file 1 — Supplementary Material [file ACM2-17-272-s001.png]

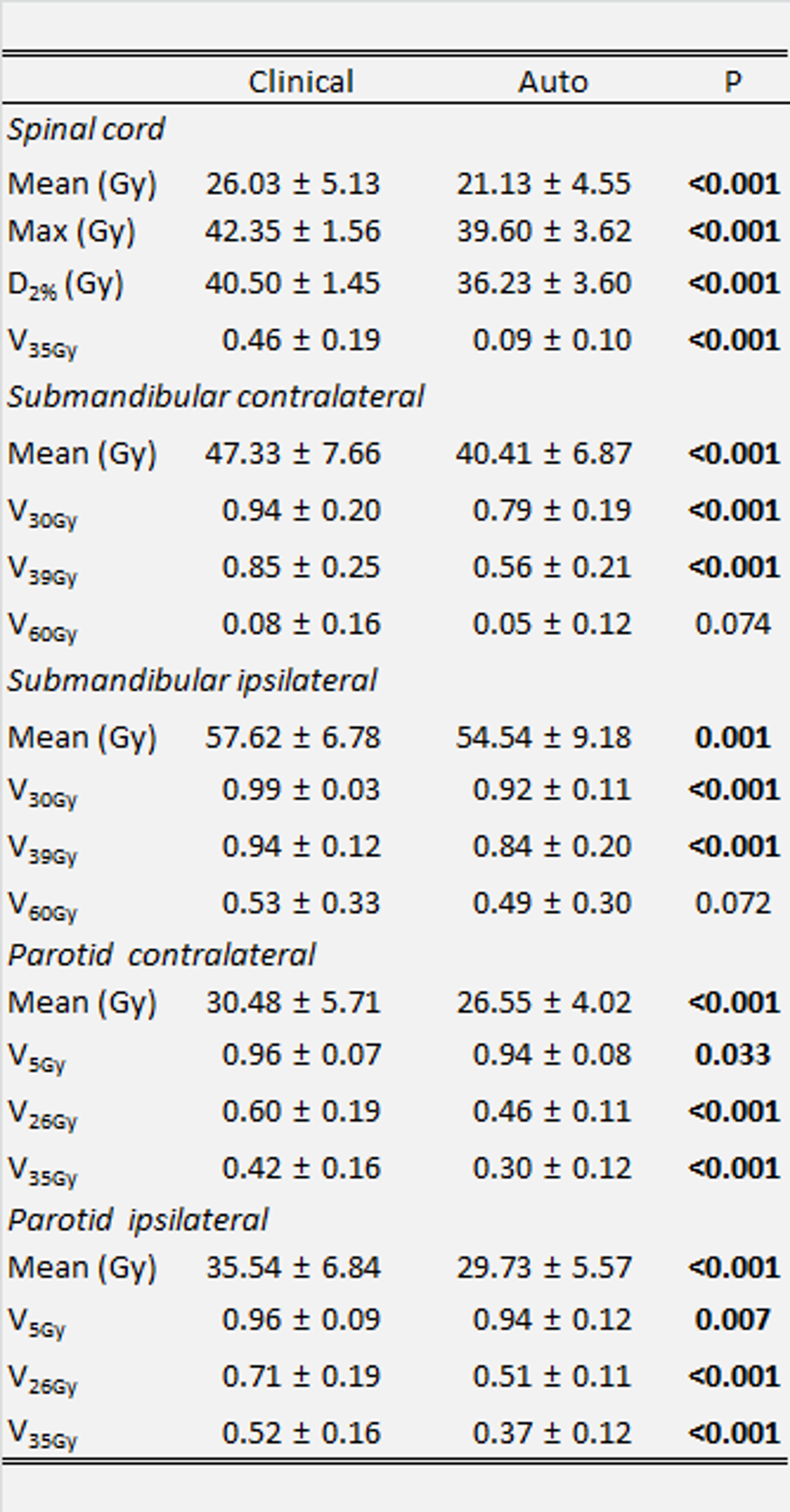

Supplement: Supplementary file 2 — Supplementary Material [file ACM2-17-272-s002.png]

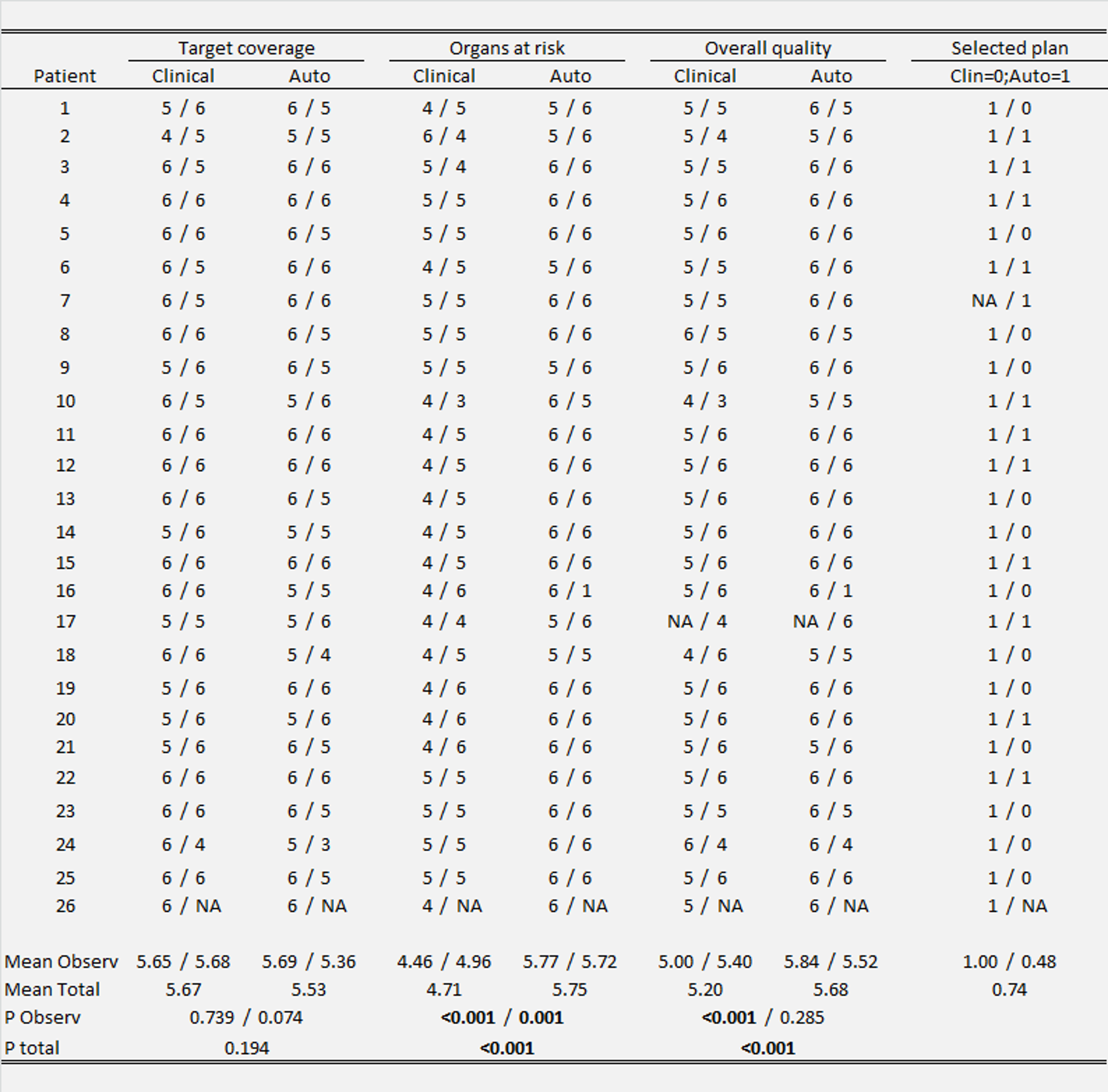

Supplement: Supplementary file 3 — Supplementary Material [file ACM2-17-272-s003.png]
